# Placenta Prævia Treated by Prof. Simpson’s Method

**Published:** 1846-01

**Authors:** J. Jones


*Placenta Prævia treated by Professor Simpson's Method.* By J. Jones, Esq.—Having read Professor Simpson's interesting paper on placental presentations, wherein he recommends extraction of the placenta before the birth of the child, I determined to give the practice a trial the first opportunity that presented itself. I soon happened to have the following case, which appears to me to prove satisfactorily, as far as can be judged from an isolated case, the superiority of the new over the usual mode of procedure under these appaling circumstances; at least, inasmuch as the safety of the mother, which should be the chief object of our solicitude, is concerned.

I was summoned, on the 14th of March, to a poor woman in labor of her sixth child, who was represented as having profuse hæmorrhage, which had continued almost interruptedly for about four hours. On my arrival, I found my patient in a most precarious state from exhaustion, with a pulse averaging from 130 to 140; she was almost speechless, slightly delirious, and there was no uterine action. There was then no hæmorrhage. I was informed she had had repeated slight attacks of hæmorrhage for six weeks previously. Having rallied her with twenty drops of tincture of opium combined with weak brandy and water, I proceeded to make an internal examination. Before I could exactly ascertain the state of the parts, I was deluged with a frightful gush of blood. The os uteri was fully dilated, the membranes entire, the presentation of the head natural: and the placenta covered nearly two-thirds of the os uteri. The hæmorrhage persisting, and there being now considerable uterine pains, I saw no time was to be lost. I accordingly, and without the least difficulty, insinuated my fingers between the adherent portion of the placenta and the uterus, and in less than a minute, the latter was freed of the placenta. I now administered a full dose of the tincture of ergot, which brought on strong expulsive pains, and the woman was delivered of a still-born child in one hour and thirty-five minutes after my entering the house. After the placenta had been extracted, I do not think she lost altogether, more than a teacupful of blood; she recovered as soon as females generally do after an ordinary labor, excepting that she continued unusually weak for a considerable time, as was to be expected after the great loss of blood. She had had one similar labor before; the the child was then turned with great difficulty (her pelvis not being capacious), peritonitis ensued, and she very narrowly escaped with her life, after a confinement to her bed of more than two months.

This case needs no comment; the facts speak for themselves. To me it is convincing proof the expediency of this mode of treatment in preference to the old one.—*London Lancet.*

